# Improving Biomethanation of Chicken Manure by Co-Digestion with Ethanol Plant Effluent

**DOI:** 10.3390/ijerph16245023

**Published:** 2019-12-10

**Authors:** Dae-Yeol Cheong, Jeffrey Todd Harvey, Jinsu Kim, Changsoo Lee

**Affiliations:** 1 GeoSynFuels, LLC, Golden, CO 80401, USAtharvey@global-resource-eng.com (J.T.H.); 2 School of Urban and Environmental Engineering, Ulsan National Institute of Science and Technology (UNIST), Ulsan 44919, Korea; penguin0529@unist.ac.kr

**Keywords:** anaerobic digestion, chicken manure, co-digestion, co-substrate, ethanol plant effluent

## Abstract

As the global production of chicken manure has steadily increased, its proper management has become a challenging issue. This study examined process effluent from a bioethanol plant as a co-substrate for efficient anaerobic digestion of chicken manure. An anaerobic continuous reactor was operated in mono- and co-digestion modes by adding increasing amounts of the ethanol plant effluent (0%, 10%, and 20% (v/v) of chicken manure). Methanogenic performance improved significantly in terms of both methane production rate and yield (by up to 66% and 36%, respectively), with an increase in organic loading rate over the experimental phases. Correspondingly, the specific methanogenic activity was significantly higher in the co-digestion sludge than in the mono-digestion sludge. The reactor did not suffer any apparent process imbalance, ammonia inhibition, or nutrient limitation throughout the experiment, with the removal of volatile solids being stably maintained (56.3–58.9%). The amount of ethanol plant effluent appears to directly affect the rate of acidification, and its addition at ≥20% (v/v) to chicken manure needs to be avoided to maintain a stable pH. The overall results suggest that anerobic co-digestion with ethanol plant effluent may provide a practical means for the stable treatment and valorization of chicken manure.

## 1. Introduction

Chicken manure (CM) is a potential source of many environmental problems, and its proper management is a challenging task. If discharged without proper treatment, CM can cause serious environmental problems, such as odor, pathogen release, attraction of pests, and contamination of soil and water bodies [1,2]. Various technologies, such as land application, refeeding to animals, and composting, have been applied for the treatment of CM [3]. Combustion can be a feasible and reliable way to treat CM, particularly when coupled with energy recovery. A recent study demonstrated the production of electricity from the fluidized bed combustion of CM, with the subsequent recycling of the ash as a soil conditioner or mineral fertilizer, in a full-scale installation [4]. This process was suggested to be a sustainable alternative to direct land application as it can reduce the use of fossil fuels and save emissions. Anaerobic digestion (AD) is also considered an attractive option for the treatment of CM and other high-strength organic wastes because of its ability to degrade organic pollutants while producing methane-rich biogas [5]. This characteristic makes AD a promising technology for sustainable energy production. Additionally, digestate can be used as a fertilizer or soil amendment, which can be a substantial benefit in rural areas [3]. Highly organic and nutrient-rich CM is an attractive feedstock for biogas production [6]. However, mono-digestion of CM as a sole feedstock often fails to achieve and maintain stable AD performance due to nutrient imbalance, particularly between carbon (C) and nitrogen (N) [7]. The low C/N ratio of CM, ranging from 7–10 [8], is inhibitory to microbial growth and can lead to significant deterioration of methanogenic performance [9]. Ammonia is released from the degradation of nitrogen-containing compounds, such as protein and urea, and accumulates in the form of free ammonia or ammonium ions, according to pH and temperature, in AD environments. Free ammonia is particularly toxic, and its excessive accumulation over 100 mg NH_3_-N/L should be avoided to maintain stable AD [10].

A simple and effective approach to overcome such limitations of mono-digestion is adding a co-substrate that can amend the chemical properties of the substrate mixture [11]. Co-digestion offers several advantages over mono-digestion for stable process operation by, for example, correcting nutrient imbalance, improving buffering capacity, and/or diluting inhibitors [12,13]. Of note is that co-substrate addition can have not only positive but also negative effects on AD performance or the quality of biogas and digestate [14]. Therefore, selecting a suitable co-substrate and mixing ratio is critical for successful co-digestion, and many studies have examined different substrate mixtures for enhanced biomethanation of difficult substrates, such as sewage sludge, animal manure, and spent coffee grounds [15,16,17]. Positive effects of co-digesting CM with other organic wastes on biogas production have been demonstrated in previous studies, particularly with carbon-rich biomass which can improve the C/N balance, for example, corn stover [18], maize silage [19], wheat straw [20], and apple pulp [21]. Moreover, readily biodegradable co-substrates which can boost hydrolysis and fermentation activities, for example, food waste [22] and cheese whey [23], have been used to improve the utilization of particulate organic matter in CM.

Byproducts from an ethanol production plant, rich in readily utilizable organic matter with a low nitrogen content, seem to have a strong potential as co-substrates for co-digestion with CM [24]. Global ethanol production increased two-fold from 13–27 billion gallons from 2007 to 2017 (Alternative Fuels Data Center; https://afdc.energy.gov/data). Thus, how to treat fermentation residues has become a pressing challenge as the world ethanol market has continued to grow, and valorizing the fermentation residues to biogas through co-digestion with CM can help to improve the sustainability and economy of ethanol production. However, little has been researched on the co-digestion of CM and bioethanol residues, particularly in continuous operation. To address this gap, this study investigated the effect and feasibility of adding process effluent from a bioethanol plant (BPE) as a co-substrate for the continuous anaerobic treatment of CM. A 39 L reactor was run in mono- and co-digestion modes with CM as the base substrate. BPE was added to the substrate mixture in different amounts during the co-digestion experiment to assess the effects of different substrate characteristics and compositions. The results of this study provide a useful reference for the implementation of co-digestion for CM treatment.

## 2. Materials and Methods

### 2.1. Substrate and Inoculum

Fresh CM was collected from an egg farm (Hudson, CO, USA) and sieved through a 3.3 mm mesh. The sieved CM was divided into equal portions (1.7 kg wet weight) and stored at −18 °C until use. The frozen CM was thawed at 4 °C for 1 day before use and diluted at a ratio of 1:4 (v/v) with tap water. The diluted CM was used as the base substrate for the co-digestion experiments. BPE was collected from a pilot-scale ethanol plant using lignocellulosic biomass hydrolysates as feedstock (GeoSynFuels, Golden, CO, USA). The characteristics of the substrates used in this study are presented in Table 1. The anaerobic sludge used as the inoculum was taken from a full-scale digester at a wastewater treatment plant (Littleton, CO, USA) and sieved through a 2.5 mm mesh. The total solids (TS) content of the inoculum was 27.8 g/L, with 70.5% of it being volatile solids (VS).

### 2.2. Reactor Start-Up and Operation

A continuously stirred anaerobic tank reactor with a 39 L working volume was operated at 35 ± 1 °C. The reactor pH was maintained above 7.0 with a 1:1 (v/v) mixture of 5 N NaOH and 5 N KOH solutions throughout the experiment. The reactor was installed with a wet tip gas meter (Wet Tip Gas Meter Co., Nashville, TN, USA) to measure biogas production.

The reactor was initially filled with the inoculum and tap water (25:75, v/v) and starved for two weeks. Synthetic wastewater containing glucose as the main carbon source was then added to each reactor at a concentration of 5.0 g chemical oxygen demand (COD)/L in batch mode. The stock solution of synthetic wastewater of 100 g COD/L contained (per L): 85.0 g glucose, 8.0 g meat extract, 26.0 g NH_4_Cl, 1.50 g CaCl_2_·2H_2_O, 1.25 g FeCl_2_·4H_2_O, 1.25 g MgSO_4_·7H_2_O, 5.03 g K_2_HPO_4_, and 1.00 g Na_2_S·9H_2_O. To each liter of the prepared wastewater, 1 mL of a trace element stock solution was added with the following composition (per L): 50 mg H_3_BO_3_, 500 mg MnSO_4_·H_2_O, 50 mg (NH_4_)_6_Mo_7_O_24_·4H_2_O, 50 mg AlCl_3_, 50 mg NiCl_2_, 50 mg CoCl_2_·6H_2_O, 50 mg ZnCl_2_, 30 mg CuCl_2_, and 1 mL HCl (36%, w/w). The stock solutions were kept at 4 °C. The synthetic wastewater had a COD:N:P ratio close to 300:5:1, which is often recommended for anaerobic culture and biogas production [25]. Once the biogas production was observed, semi-continuous operation started. The reactor was operated with daily feeding of the synthetic wastewater at an organic loading rate (OLR) of 1.5 g COD/L/day for over a month until stable performance was reached.

After the start-up operation with the synthetic wastewater for microbial enrichment, the reactor started to be fed with CM as the sole substrate at a feeding frequency of four times a day (set as day 0). The reactor was operated at a hydraulic retention time (HRT) of 19.5 days during the mono-digestion for more than five turnovers of the working volume (Phase I) and then run in co-digestion mode at shorter HRTs (Phases II and III) (Table 2). CM was used as the base substrate during Phases II and III in the same amount as for the mono-digestion experiment. BPE was added at 10% and 20% (v/v) of CM during Phases II and III, respectively, to investigate the effects of different substrate characteristics and compositions. The HRT and OLR were changed according to the increase in the addition of BPE over the experimental phases. The reactor pH was not controlled during Phases I to III.

### 2.3. Specific Methanogenic Activity Test

Specific methanogenic activity (SMA) was determined in duplicate using serum bottles of a working volume of 130 mL as described previously [26]. The sludge biomass for the SMA assay was taken at the end of the mono- and co-digestion phases. Each biomass was tested with different substrates at 2000 mg COD/L: glucose, acetate, propionate, *n*-butyrate, a mixture of acetate and propionate (2:1 molar ratio), and a mixture of acetate, propionate, and *n*-butyrate (2:1:1 molar ratio). Inorganic nutrients and trace mineral solutions were added for the growth of anaerobic microorganisms. The SMA culture media were initially neutralized with 10 N NaOH or 10 N HCl solutions, and NaHCO_3_ was added as necessary to maintain a neutral pH.

The kinetics of methane production were evaluated using a modified Gompertz model (Equation (1)):

(1) $$M_{t}=M_{P}\cdot \exp\left[-\exp\left\{\frac{R_{m}\cdot e}{M_{P}}\left(\lambda -t\right)+1\right\}\right],$$

where *M_t_* is the cumulative methane production (mL) at time *t* (d), *M_p_* is the methane production potential (mL), *R_m_* is the maximum methane production rate (mL/d), *λ* is the lag phase length (d), and *t* is the incubation time (d). SMA was determined by dividing *R_m_* by the amount of inoculum used (volatile solids).

### 2.4. Analytical Methods

Total COD (tCOD) and soluble COD (sCOD) were measured by the closed reflux colorimetric method [27]. Solids, total nitrogen (TN), and total carbon (TC) were determined as per the procedures described previously. Volatile fatty acids (VFAs, ≤C_6_) were analyzed using a gas chromatograph (GC 2014, Shimadzu, Japan) equipped with a flame ionization detector and a carbowax packed column (Supelco, Bellefonte, PA, USA), with nitrogen as the carrier gas. Samples for sCOD and VFA measurements were prepared by filtration through a glass-fiber filter (Whatman GF/C; 1.2 μm pore size). Biogas composition was determined using a gas chromatograph (SRI 8610C; Torrance, CA, USA) equipped with a thermal conductivity detector and a molecular sieve 13X packed column (Restek, Bellefonte, PA, USA), with argon as the carrier gas. Total ammonia nitrogen (TAN) and lactic acid were quantified using a 7100 MBS analyzer (YSI Life Sciences, Yellow Springs, OH, USA). Phosphorous, calcium, potassium, and sodium were quantified using an inductively coupled plasma mass spectrometer (ICP-MS; Leeman Labs Inc., Hudson, NH, USA).

## 3. Results and Discussion

**Figure 1:**
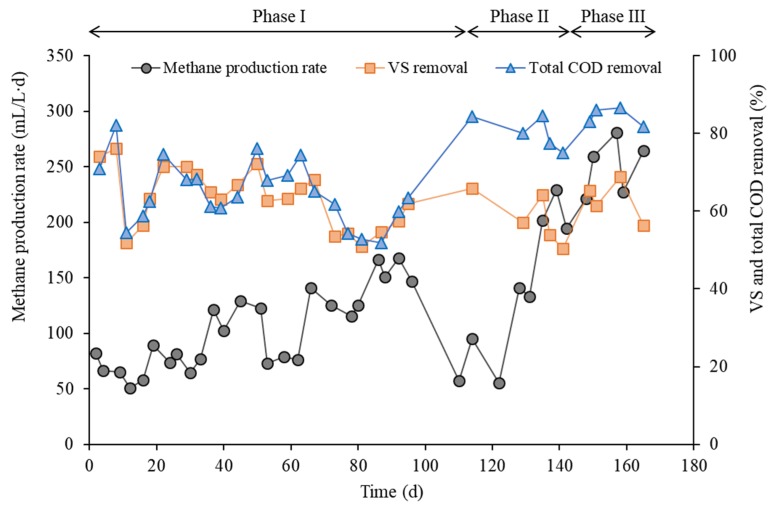
Methane production and organic removal profiles during the experimental phases.

After approximately one month of continuous mono-digestion of CM, the methane content of the biogas remained relatively stable at around 50% until the end of the experiment, regardless of the addition of BPE. Table 3 summarizes the performance of the reactor during the experimental phases. Methane production rate (MPR), that is, the volume of methane produced per unit volume of reactor per unit time, increased as the OLR increased with co-feeding of BPE (Phases II and III). The highest MPR was observed in Phase III (258 mL/L/d), which was 66% higher than that in Phase I (155 mL/L/d). This increase in MPR is attributable to the addition of a more readily utilizable co-substrate BPE. Correspondingly, tCOD removal efficiency also increased over the experimental phases. Meanwhile, VS removal varied little (56.2–61.5%) across the experimental phases, which can be attributed to the high tCOD/VS ratio of BPE (15 g tCOD/g VS; Table 1). The observed VS removal was comparable to previously reported data for the continuous AD of CM (30–55%) [28]. It is noteworthy that MPR fluctuated more during the co-digestion phases than during the mono-digestion phase (Figure 1), which may be related to the feeding frequency of BPE. Given its high COD content, semi-daily feeding of BPE may have caused temporary overloading that directly affected the AD performance. This result suggests that the effect of feeding regime needs to be considered for stable operation of a co-digestion process when feeding a co-substrate with high organic content. Applying a higher feeding frequency or fully continuous feeding of BPE may help to reduce the fluctuations in methane production during co-digestion with CM [29,30]. The methane yield per VS fed (Y_Mf_) in Phase I was 396 mL/g VS, which corresponds to the values reported for the mono-digestion of CM in other studies (200–400 mL/g VS) [1,28,31]. With the addition of BPE over the following co-digestion phases, Y_Mf_ increased significantly to 478 mL/g VS fed in Phase II and then to 540 mL/g VS fed in Phase III. The methane yield per VS removed (MY_r_) also increased correspondingly over the co-digestion phases (Table 3). These results show that digesting CM with BPE as a co-substrate could enhance the anaerobic conversion of organic solids in the substrate mixture.

**Figure 2:**
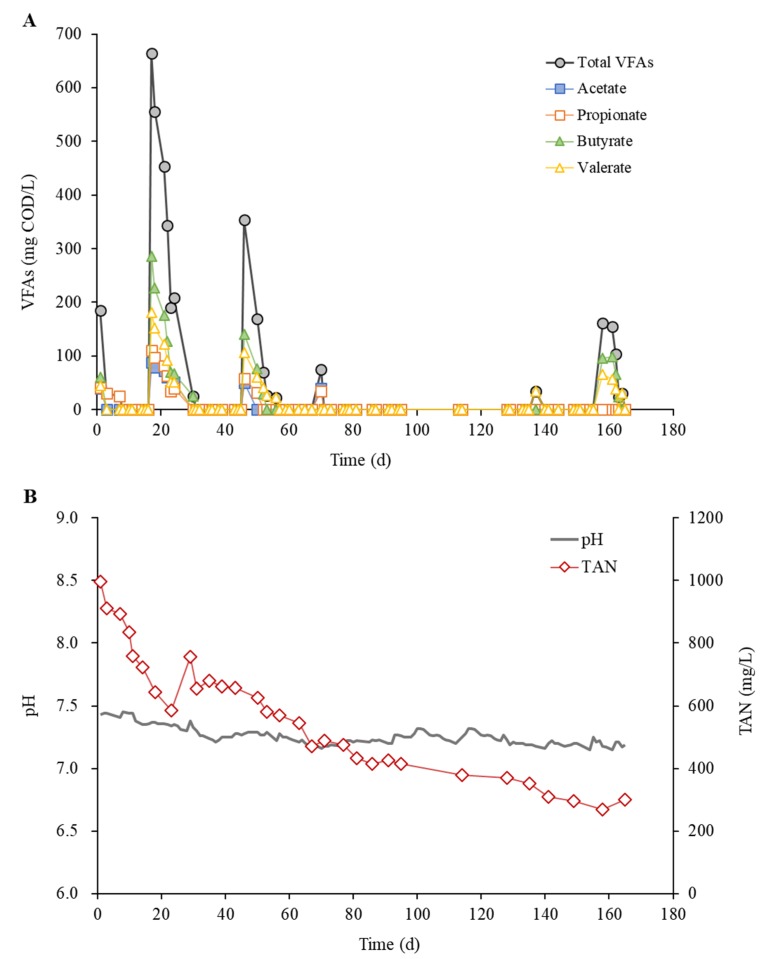
Residual concentrations of volatile fatty acids (VFAs) (**A**) and total ammonia nitrogen (TAN) (**B**).

Acetate, propionate, butyrate, and valerate were the major intermediate VFAs, and their total concentration remained at low levels (<200 mg/L as COD) after approximately two months of CM mono-digestion and until the end of the experiment (Figure 2A). Lactate was not detected throughout the experiment (data not shown), which can be attributed to the rapid conversion of lactate by diverse bacteria to VFAs, particularly acetate and propionate, in anaerobic environments [32]. Although small amounts of VFAs accumulated temporarily during the experimental phases, their concentration profiles were generally stable, especially during Phases II and III. Correspondingly, the reactor pH remained in a neutral range throughout the experiment without pH control, except for Phase III where an alkaline solution (see Section 2.2) was added to maintain the pH above 7.0 (Figure 2B). These results suggest that BPE can be co-fed up to 10% (v/v) with CM for the stable co-digestion of the mixture under uncontrolled pH conditions. The need for pH control during Phase III indicated that, unless the reactor pH was controlled, co-feeding BPE at 20% (v/v) with CM would lead to a process imbalance or reactor souring. The explanation for this can be that the increased loading of readily biodegradable organic matter promotes fermentation and potentially causes an imbalance between the production and consumption of organic acids [33]. The use of buffer chemicals for pH control has drawbacks such as additional operating costs and accumulation of potentially toxic salts [32]. Therefore, the mixing ratio between CM and BPE is an important factor to consider for their stable and cost-effective co-digestion under uncontrolled pH conditions. Of note here is that the amount of BPE co-fed with CM directly affects the acidification rate, and the co-digestion system may be upset at a BPE-to-CM ratio between 10% and 20% (v/v). Due to the nature of the experimental design, it was not possible to determine in this study whether digester acidification occurs at a BPE-to-CM ratio of 20% (v/v) or at a lower percentage.

The TAN concentration gradually decreased from 1000 mg/L to 400 mg/L during Phase I and then remained at 300 mg/L to 400 mg/L during Phases II and III (Figure 2B). TAN exists predominantly as NH_4_^+^-N under the pH and temperature conditions of the reactor, and the concentration of free ammonia (NH_3_-N) was estimated to be less than 20 mg/L throughout the experiment. This is well below the threshold above which methanogenic activity can be significantly inhibited [10]. Meanwhile, the NH_4_^+^-N concentration remained well above the required minimum of 40–70 mg/L for stable methanogenic activity [10] throughout the experiment. These results suggest that there was neither ammonia inhibition nor nitrogen limitation in the reactor. Given that ammonia acts as a buffer against acidification, residual ammonia, liberated primarily from proteins and urea in CM, likely also contributed to the maintenance of neutral pH.

The SMA values of the reactor biomass, taken during Phases I and III for six different carbon sources, are presented in Table 4. The SMA test is used to assess the substrate-dependent methanogenic activity and viability of microbial biomass. Each biomass showed generally comparable results for the VFAs and VFA mixtures used for the SMA tests, with the SMA for glucose being significantly lower. This difference is attributable to the consumption of carbon and energy for the fermentative conversion of glucose to VFAs. It is worth noting that the co-digestion biomass from Phase III had significantly higher SMA values (by 24.6–68.7%) than the mono-digestion biomass from Phase I for all substrates tested. This result corresponds to the increased methane production rate and yield over the experimental phases with the addition of BPE (Table 3), further supporting the enhancing effect of the co-digestion of CM with BPE on methanogenic activity.

The overall results suggest that CM can be effectively and stably co-digested with BPE. The reactor performance was enhanced in terms of both methane production rate and yield with the addition of BPE as a co-substrate. BPE is highly rich in carbohydrates, including residual sugars from ethanol fermentation, and the beneficial effect of co-digestion seems to be largely due to the increase in readily utilizable organic carbon, whereby the reactor microbial activity, and also the substrate C/N ratio, could be improved. Many previous studies have examined the anaerobic co-digestion of CM with other organic wastes and reported beneficial effects of co-digestion on methane production. However, as described in the introduction, most of these studies have tested agricultural residues with a high C/N ratio (i.e., adjustment of substrate C/N ratio) [18,19,20,21] or readily biodegradable food and food-processing wastes (i.e., enhanced hydrolysis of particulate organic matter) [22,23] as co-substrates, and there is little literature on the use of BPE as a co-substrate for the AD of CM. Moreover, the majority of previous studies were conducted in batch mode, and limited information is available on the anaerobic treatment of CM through co-digestion in continuous mode. Therefore, the results of the present study, which performed the anaerobic co-digestion of CM with BPE in continuous mode, will provide a useful addition to the literature on the management of CM and other animal manures. Co-digestion studies of CM with high C/N ratio lignocellulosic biomass, such as corn stover or maize silage, suggested a CM proportion of 20–50% (on a VS basis) in the substrate mixture for stable AD [18,19]. In this study, the experimental reactor achieved stable AD performance at CM proportions higher than 80% (on a VS basis) in continuous mode without significant ammonia inhibition, which can be attributed to the very high organic carbon content of BPE. A study which co-digested CM with readily biodegradable cheese whey in continuous mode reported a safe limit of 50% whey proportion (on a VS basis) to prevent performance deterioration due to acidification [23]. Similarly, acidification was observed in the present study during co-digestion at a BPE-to-CM ratio of 20% (v/v), which corresponds to a CM proportion of 18% (on a VS basis). These results suggest that BPE is an effective co-substrate for the anaerobic treatment of CM and can significantly improve the AD of the CM/BPE mixture even with a relatively small amount of addition.

## 4. Conclusions

Anaerobic co-digestion of CM with BPE was tested with increasing amounts of BPE addition (0%, 10%, and 20% (v/v) of CM) in continuous mode. The methane production rate and yield increased significantly by up to 66% and 36%, respectively, as a result of the increase in organic and hydraulic loading rates with the addition of BPE. Additionally, the co-digestion biomass showed significantly higher SMA values than the mono-digestion biomass for all substrates tested (i.e., acetate, propionate, *n*-butyrate, mixed VFAs, and glucose). There was no sign of process imbalance, ammonia inhibition, or nutrient limitation throughout the experiment. The overall results suggest that BPE is a suitable co-substrate for effective treatment and valorization of CM, and potentially other animal manures, through AD.

## Figures and Tables

**Table 1:** Physicochemical characteristics of the substrates used.

Parameter	Chicken Manure	BPE ^a^
Total solids (g/L)	13.2–28.9	26.4
Volatile solids (g/L)	5.5–9.1	7.5
Total carbon (g/L)	39 (<1.0) ^b^	− ^c^
Total nitrogen (g/L)	4 (<1.0)	−
C/N ratio	9.75 (<1.0)	−
pH	6.9–7.4	5.3–5.4
Total chemical oxygen demand (g/L)	8.7–13.0	111.8
Soluble chemical oxygen demand (g/L)	3.2–9.7	110.3
Total ammonia nitrogen (mg/L)	150–360	−
Total volatile fatty acids (mg COD/L)	100–500	2541
Acetate (mg/L)	60–300	2264
Propionate (mg/L)	25–140	30
*n*-Butyrate (mg/L)	20–50	40
*n*-Valerate (mg/L)	20–60	not detected
Lactate (mg/L)	3–20	184
Phosphorous (mg/L)	81 (<1.0)	−
Calcium (mg/L)	206 (<1.0)	−
Potassium (mg/L)	547 (<1.0)	−
Sodium (mg/L)	271 (<1.0)	−

^a^ Process effluent from a bioethanol plant (BPE). ^b^ Relative standard deviation (%) of one representative sample is given in parentheses (*n* > 3). ^c^ Not determined. COD: chemical oxygen demand.

**Table 2:** Reactor operating conditions during the experimental phases.

Parameter ^a^	Mono-Digestion	Co-Digestion	
	Phase I	Phase II	Phase III
Period (d)	0–112	112–143	143–165
CM feeding rate (L/d)	2.0	2.0	2.0
BPE feeding rate (L/d)	– ^b^	0.2	0.4
CM feeding frequency (times/d)	4	4	4
BPE feeding frequency (times/d)	–	2	2
Hydraulic retention time (d)	19.5	17.7	16.3
Organic loading rate (g tCOD/L/d)	0.65	1.13	1.80
Organic loading rate (g VS/L/d)	0.39	0.42	0.47

^a^ CM: chicken manure; BPE: process effluent from a bioethanol plant; tCOD: total COD; VS: volatile solids. ^b^ Not applicable.

**Table 3:** Reactor performance during the mono- and co-digestion phases.

Parameter	Mono-Digestion	Co-Digestion	
	Phase I	Phase II	Phase III
Data acquisition period (d)	88–96	135–142	157–165
Total COD removal (%)	54.4	79.0	83.2
Volatile solids removal (%)	58.0	56.2	61.5
Methane production rate (mL/L/d)	155	208	258
Methane yield (mL/g VS fed)	396	478	540
Methane yield (mL/g VS removed)	672	821	958

**Table 4:** Specific methanogenic activity of reactor sludge on different substrates.

Substrate ^a^	Mono-Digestion Sludge (Phase I)		Co-Digestion Sludge (Phase III)	
	R_m_^b^ (mL/d)	SMA ^c^ (mL/g VS/d)	R_m_ (mL/d)	SMA (mL/g VS/d)
Glucose	4.20	2.17	5.17	3.66
HAc	8.18	4.23	7.44	5.27
HPr	8.16	4.22	8.72	6.18
HBu	6.98	3.61	7.56	5.35
HAc:HPr (2:1)	6.72	3.48	8.09	5.73
HAc:HPr:HBu (2:1:1)	7.49	3.88	7.85	5.56

^a^ HAc: acetate; HPr: propionate; Hbu: butyrate; molar mixing ratios are in parentheses. ^b^ Maximum methane production rate (R_m_). ^c^ Specific methanogenic activity (SMA).

## References

[1] Sakar S., Yetilmezsoy K., Kocak E. (2009). Anaerobic digestion technology in poultry and livestock waste treatment—A literature review. Waste Manag. Res..

[2] Wang F., Pei M., Qiu L., Yao Y., Zhang C., Qiang H. (2019). Performance of anaerobic digestion of chicken manure under gradually elevated organic loading rates. Int. J. Environ. Res. Public Health.

[3] Bolan N.S., Szogi A.A., Chuasavathi T., Seshadri B., Rothrock M.J., Panneerselvam P. (2010). Uses and management of poultry litter. World’s Poult. Sci. J..

[4] Billen P., Costa J., Van der Aa L., Van Caneghem J., Vandecasteele C. (2015). Electricity from poultry manure: A cleaner alternative to direct land application. J. Clean. Prod..

[5] Manyi-Loh C.E., Mamphweli S.N., Meyer E.L., Okoh A.I., Makaka G., Simon M. (2013). Microbial anaerobic digestion (bio-digesters) as an approach to the decontamination of animal wastes in pollution control and the generation of renewable energy. Int. J. Environ. Res. Public Health.

[6] Rajagopal R., Massé D.I. (2016). Start-up of dry anaerobic digestion system for processing solid poultry litter using adapted liquid inoculum. Process Saf. Environ. Prot..

[7] Duan N., Ran X., Li R., Kougias P.G., Zhang Y., Lin C., Liu H. (2018). Performance evaluation of mesophilic anaerobic digestion of chicken manure with algal digestate. Energies.

[8] Marchioro V., Steinmetz R.L.R., do Amaral A.C., Gaspareto T.C., Treichel H., Kunz A. (2018). Poultry litter solid state anaerobic digestion: Effect of digestate recirculation intervals and substrate/inoculum ratios on process efficiency. Front. Sustain. Food Syst..

[9] Molaey R., Bayrakdar A., Sürmeli R.Ö., Çalli B. (2018). Anaerobic digestion of chicken manure: Mitigating process inhibition at high ammonia concentrations by selenium supplementation. Biomass Bioenergy.

[10] Speece R.E. (1996). Anaerobic Biotechnology for Industrial Wastewaters.

[11] Fonoll X., Astals S., Dosta J., Mata-Alvarez J. (2015). Anaerobic co-digestion of sewage sludge and fruit wastes: Evaluation of the transitory states when the co-substrate is changed. Chem. Eng. J..

[12] Esposito G., Frunzo L., Giordano A., Liotta F., Panico A., Pirozzi F. (2012). Anaerobic co-digestion of organic wastes. Rev. Environ. Sci. Biotechnol..

[13] Mata-Alvarez J., Dosta J., Romero-Güiza M.S., Fonoll X., Peces M., Astals S. (2014). A critical review on anaerobic co-digestion achievements between 2010 and 2013. Renew. Sustain. Energy Rev..

[14] Xie S., Higgins M.J., Bustamante H., Galway B., Nghiem L.D. (2018). Current status and perspectives on anaerobic co-digestion and associated downstream processes. Environ. Sci. Water Res. Technol..

[15] Nghiem L.D., Koch K., Bolzonella D., Drewes J.E. (2017). Full scale co-digestion of wastewater sludge and food waste: Bottlenecks and possibilities. Renew. Sustain. Energy Rev..

[16] O’Brien B.J., Milligan E., Carver J., Roy E.D. (2019). Integrating anaerobic co-digestion of dairy manure and food waste with cultivation of edible mushrooms for nutrient recovery. Bioresour. Technol..

[17] Kim J., Baek G., Kim J., Lee C. (2019). Energy production from different organic wastes by anaerobic co-digestion: Maximizing methane yield versus maximizing synergistic effect. Renew. Energy.

[18] Li Y., Zhang R., Chen C., Liu G., He Y., Liu X. (2013). Biogas production from co-digestion of corn stover and chicken manure under anaerobic wet, hemi-solid, and solid state conditions. Bioresour. Technol..

[19] Sun C., Cao W., Banks C.J., Heaven S., Liu R. (2016). Biogas production from undiluted chicken manure and maize silage: A study of ammonia inhibition in high solids anaerobic digestion. Bioresour. Technol..

[20] Hassan M., Umar M., Ding W., Mehryar E., Zhao C. (2017). Methane enhancement through co-digestion of chicken manure and oxidative cleaved wheat straw: Stability performance and kinetic modeling perspectives. Energy.

[21] Li K., Liu R., Cui S., Yu Q., Ma R. (2018). Anaerobic co-digestion of animal manures with corn stover or apple pulp for enhanced biogas production. Renew. Energy.

[22] Wang M., Sun X., Li P., Yin L., Liu D., Zhang Y., Li W., Zheng G. (2014). A novel alternate feeding mode for semi-continuous anaerobic co-digestion of food waste with chicken manure. Bioresour. Technol..

[23] Gelegenis J., Georgakakis D., Angelidaki I., Mavris V. (2007). Optimization of biogas production by co-digesting whey with diluted poultry manure. Renew. Energy.

[24] Cassidy D.P., Hirl P.J., Belia E. (2008). Methane production from ethanol co-products in anaerobic SBRs. Water Sci. Technol..

[25] Leite W., Magnus B.S., Guimarães L.B., Gottardo M., Belli Filho P. (2017). Feasibility of thermophilic anaerobic processes for treating waste activated sludge under low HRT and intermittent mixing. J. Environ. Manag..

[26] Soto M., Méndez R., Lema J. (1993). Methanogenic and non-methanogenic activity tests. Theoretical basis and experimental set up. Water Res..

[27] Rice E.W., Baird R.B., Eaton A.D. (2005). Standard Methods for the Examination of Water and Wastewater.

[28] Nie H., Jacobi H.F., Strach K., Xu C., Zhou H., Liebetrau J. (2015). Mono-fermentation of chicken manure: Ammonia inhibition and recirculation of the digestate. Bioresour. Technol..

[29] Nachaiyasit S., Stuckey D.C. (1997). The effect of shock loads on the performance of an anaerobic baffled reactor (ABR). 2. Step and transient hydraulic shocks at constant feed strength. Water Res..

[30] Cheong D.-Y., Hansen C.L. (2008). Effect of feeding strategy on the stability of anaerobic sequencing batch reactor responses to organic loading conditions. Bioresour. Technol..

[31] Niu Q., Qiao W., Qiang H., Hojo T., Li Y.-Y. (2013). Mesophilic methane fermentation of chicken manure at a wide range of ammonia concentration: Stability, inhibition and recovery. Bioresour. Technol..

[32] Jo Y., Kim J., Hwang K., Lee C. (2018). A comparative study of single- and two-phase anaerobic digestion of food waste under uncontrolled pH conditions. Waste Manag..

[33] Nielsen H.B., Uellendahl H., Ahring B.K. (2007). Regulation and optimization of the biogas process: Propionate as a key parameter. Biomass Bioenergy.

